# Determination of *Alternaria* Toxins in Sunflower Oil by Liquid Chromatography Isotope Dilution Tandem Mass Spectrometry

**DOI:** 10.3390/molecules25071685

**Published:** 2020-04-07

**Authors:** Ádám Tölgyesi, Luca Kozma, Virender K. Sharma

**Affiliations:** 1 KERMI Department, ÉMI-TÜV SÜD Ltd., Dugonics utca 11, 1043 Budapest, Hungary; kozma.luca@emi-tuv.hu; 2 Program for the Environment and Sustainability, Department of Environmental and Occupational Health, School of Public Health, Texas A&M University, 212 Adriance Lab Rd., 1266 TAMU, College Station, TX 77843, USA

**Keywords:** *Alternaria* toxins, LC-MS/MS, isotope dilution, sunflower oil, validation, real sample analysis

## Abstract

*Alternaria* toxins have gained attention as a potential health risk and can be classified as emerging mycotoxins. As a result, they are candidates to be regulated by the European Commission. This paper describes a liquid chromatography tandem mass spectrometric (LC-MS/MS) method for analyzing five *Alternaria* toxins in sunflower oil, which is a rather different type of sample to those matrices investigated in earlier published papers. An optimal sample preparation condition was achieved when samples were dissolved in *n*-hexane and extracted with methanol/water mixture, followed by sample pre-concentration with solvent evaporation. This study is the first focusing only on this lipophilic matrix and in using all corresponding isotopically labeled internal standards (ISTD) to compensate the matrix effect that strongly influences the LC-MS/MS analysis of toxins. Target compounds were separated on Zorbax Extend C-18 column enabling the analysis at alkaline pH of 8.8 that was necessary to obtain appropriate peak shape of tenuazonic acid and to separate the analytes at baseline. The method was validated according to the EU 2002/657/EC Decision and all the analytical performance characteristics met the requirements. The recovery was between 74% and 122% in fortified sunflower oil samples and the precision varied from 9% to 22%. The method was successfully demonstrated for sunflower seed quality check (QC) samples. Finally, 16 different sunflower oil samples were measured; and tenuazonic acid and tentoxin toxins were detected at levels close to LOQ concentrations.

## 1. Introduction

**Figure 1:**
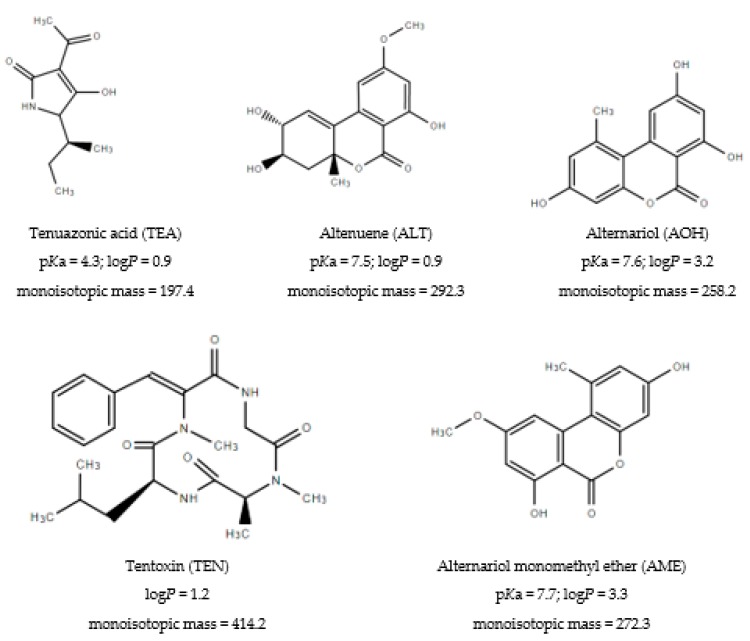
Structure and physical-chemical properties of five toxins analyzed in this study.

Clear evidence of animal and human illness and death caused by fungal metabolites have been reported worldwide since the 1970s. The secondary metabolites of fungi growing on agricultural commodities, called mycotoxins, are still considered as a major health concern [1]. Analytical methods, therefore, have been developed and subsequently validated to determine mycotoxins in different food and feed samples [2]. These methods are used in the monitoring laboratories to screen and confirm the samples that are contaminated with mycotoxins. In the European Union (EU), the maximum levels (ML) of regulated mycotoxins in food are in force [3]. For some mycotoxins without any ML, the European Food Safety Authority (EFSA) has published opinions, often indicating the need of harmonized and suitable methods to make sound exposure assessments. According to the EFSA, cereals, vegetables, and oilseeds frequently contain *Alternaria* mycotoxins, which could cause mutagenic, genotoxic, fetotoxic, and teratogenic effects [4]. These agricultural commodities are mostly infected by *Alternaria* species (e.g., *Alternaria alternata*) that produce more than 70 secondary metabolites from which the five most important ones are tenuazonic acid (TEA), altenuene (ALT), alternariol (AOH), tentoxin (TEN), and alternariol monomethyl ether (AME) (Figure 1) [4]. Currently, these *Alternaria* toxins are not regulated because there has not been enough information available to establish ML (i.e., risk assessment). Additionally, the development of a standard method is imperative, which is not available yet [4]. Hence, a few control laboratories have analyzed them on a regular basis using LC-MS/MS technique, which is the most suitable technique according to the EFSA [4]. However, interlaboratory comparisons (ILC) have already been organized for these toxins in order to support the legislation [5,6].

The first ILC was a proficiency test (PT) for *Alternaria* toxins in tomato juice, organized by the Federal Institute for Risk Assessment (BfR, Berlin, Germany) in 2014 [5]. Afterwards, the European Commission Joint Research Centre, EU Reference Laboratory for Mycotoxins (JRC, Geel, Belgium), performed the method validation study (MVS) to find a candidate LC-MS/MS method as a possible basis for drafting a standard method for *Alternaria* toxins in 2015 [6]. The MVS was started with a pre-trial and included tomato juice as test samples, followed by the final trial with tomato juice, cereals and sunflower seed samples. The reproducibility of the candidate method did not fulfill the requirements of the European Committee for Standardization (CEN). Due to lack of isotopically labeled internal standards (ISTDs), the candidate method showed low interlaboratory precision of some compounds (i.e., TEA and AME) [6]. In 2018, the ISTDs for five *Alternarias* mentioned above became commercially available. Therefore, the MVS was repeated by utilizing the LC-MS/MS determination with isotope dilution (LC-ID-MS/MS) and the obtained results met the requirements [7]. This MVS showed that the application of isotope dilution is critically important for analyzing *Alternaria* toxins in food samples using LC-MS/MS method.

Furthermore, Liu and Rychlik [8] published the advantage of using isotopically labeled TEN and its derivates for quantification of toxins in various food samples. They reported the synthesis and application of TEN-d3, DH-TEN-d3 (dihydrotentoxin-d3), and isoTEN-d3 (isotentoxin-d3) for quantifying native toxins in cereal-, vegetable-, and fruit-based samples and included different types of oils as well [8]. In another research performed by Liu and Rychlik, the biosynthesis of ^13^C-labeled ISTDs for seven *Alternaria* toxins was described [9]. The application of AOH-^13^C14, ALT-^13^C15, and AME-^13^C15 in the future can further enhance the quantification of toxins in food since the ^13^C-labeled ISTDs have advantages over the deuterated ISTDs. Namely, there is no retention time difference between the native target compound and its corresponding ^13^C-labeled analogue. This enables the total compensation of matrix effect (ME) during LC-MS/MS analysis. Furthermore, there will not be substantial overlap between ISTD signals and the isotopic signals of analyte if the molecular mass of the isotopologue is more than 5 mass units. However, the authors reported the isotope effect between AME and AME-^13^C15 when acetonitrile/2-propanol mixture was used as the organic modifier in the eluent [9]. Therefore, only the deuterated AOH and AME are commercially available so far.

This paper describes the use of a LC-ID-MS/MS method for analyzing five toxins mentioned above in sunflower oil samples for the first time. Even though the high contamination of sunflower seeds with *Alternaria* toxins (TEA: LOQ—5400 µg/kg; AOH: LOQ—1200 µg/kg; TEN: LOQ - 880 µg/kg; AME: LOQ—440 µg/kg) have been recently reported worldwide [4,6,10,11,12], the existing methods (Table S1) involved mainly the vegetable-, cereal-, fruit-based, and oilseed samples [8,9,10,11,12,13,14,15,16,17,18,19,20,21,22,23,24,25,26,27,28,29,30,31,32,33,34,35,36]. The oil samples got less attention, so far only three studies have included the analysis of this matrix [8,11,12]. The reason for excluding this sample matrix could be the different sample manipulations needed for this lipophilic sample. Thus, this matrix is now in the focus of the current study. The aims of the work presented here were to: (i) set up LC-ID-MS/MS separation process for five *Alternaria* toxins without chemical derivatization; (ii) develop a sample preparation approach, which is suitable for sunflower oil; (iii) fine-tune the LC-ID-MS/MS method to achieve the quantification limit as low as possible; (iv) perform inhouse validation of the method to meet the requirements set by EU; and (v) apply the method for real samples and also naturally contaminated and spiked sunflower seed QC samples.

## 2. Results

### 2.1. General Conditions for LC-ID-MS/MS Separation

The fine-tuning of ion transitions in the MS/MS instrument was carried out with individual standard solutions (2 µg/mL) in methanol and employing electrospray (ESI) source in negative ion mode according to our previous paper [13]. The isotopically labeled ISTDs were also tuned using the ISTD mixture (Section 4.1). The two most intense ion transitions of target compounds used for the MS/MS detection are detailed in Table 1. The ALT, AOH, and AME toxins are weak acidic molecules (Figure 1) and show an appropriate retention on C-18 HPLC columns and high sensitivity during MS or MS/MS detection [9,10,11,12,13,14,15,16,17,18,19,20,21,22,23,24,25,26,27,28,29,30,31,32,33,34,35,36]. TEN can be considered as a neutral molecule but can be also measured by LC-MS/MS method with negative ionization and fit-for-purpose sensitivity [8,10,11,12,13]. In the case of TEA, however, special HPLC conditions are necessary due to its different isomer forms appearing in aqueous phase at acidic pH [13,19,20]. Therefore, chemical derivatization with 2,4-dinitrophenylhidrazine was introduced for TEA that makes it a suitable compound for HPLC analysis [13,19,20]. The drawback of the derivatization approach is the longer sample preparation time, lower selectivity, and increased noise of analysis [13]. Recently, HPLC separations at alkaline pH conditions on C-18 column were reported for TEA separation in food matrices [6,21,22,30,31,33]. A pH above 8.0 results in reproducible retention time and peak shape for TEA but decreases its retention time due to the deprotonated hydrophilic form of TEA at alkaline pH (Figure 1).

**Figure 2:**
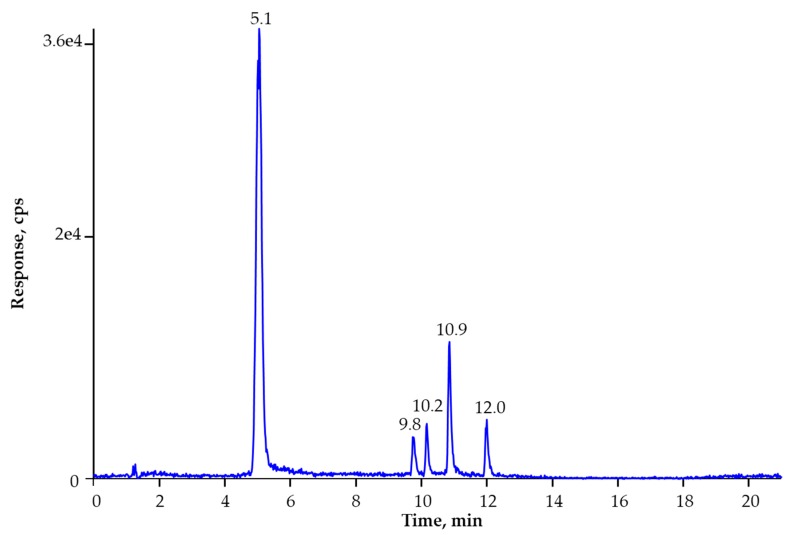
Total ion current chromatogram of five toxins at 10 µg/kg using LC-APCI(-)-MS/MS separation at pH 8.8. Compounds: TEA (5.1 min); ALT (9.8 min); AOH (10.2 min); TEN (10.9 min); and AME (12.0 min). The concentrations of ISTDs were: TEA-13C2 (83 µg/kg), ALT-d6 (33 µg/kg), AOH-d3 (17 µg/kg), TEN-d3 (17 µg/kg), and AME-d3 (17 µg/kg).

This condition, however, does not require derivatization, hence, it was tested in the present study with a HPLC column suitable for separation at above pH 8.0. A Zorbax Extend C-18 column allows separation at pH up to 11.5. The alkaline condition (Section 4.7) resulted in baseline separation for the five toxins and appropriate retention for TEA on this column (Figure 2). The pH of the mobile phase was tested between 8.0 and 9.0. Retention time shift and difference in sensitivity were not observed. The apparent retention factor (k’) calculated for TEA was higher than 2.0 under all conditions. However, the ESI source did not result in enough sensitivity for the analysis, and the instrumental limit of quantification (LOQ) was not lower than 50 ng/mL, but the aim was to detect AME and AOH below 10 ng/mL [6]. Consequently, the ESI positive ionization mode was also tested (Table 1) under acidic pH condition (Section 4.7), but a better sensitivity could not be achieved. Moreover, the peak shape of TEA was irreproducible under acidic condition using this HPLC column mentioned above. Therefore, the atmospheric pressure chemical ionization (APCI) source was also tested in negative ion mode and under alkaline HPLC condition. It should be pointed out that the same ion transitions were used for performing the detections in both ESI and APCI modes. Only different polarities (positive or negative) resulted in various ion traces. This instrument gave increased sensitivity for these toxins with APCI source using negative ionization. The instrumental LOQ could be lowered at least with one order of magnitude for all compounds in comparison to those values obtained with ESI probe. The best LC-MS/MS conditions were obtained using alkaline pH condition for HPLC separation at pH 8.8 and employing APCI source with negative ionization mode (Figure 2).

### 2.2. Development of Sample Preparation

#### 2.2.1. Sample Preparation without SPE Clean-Up

**Figure 3:**
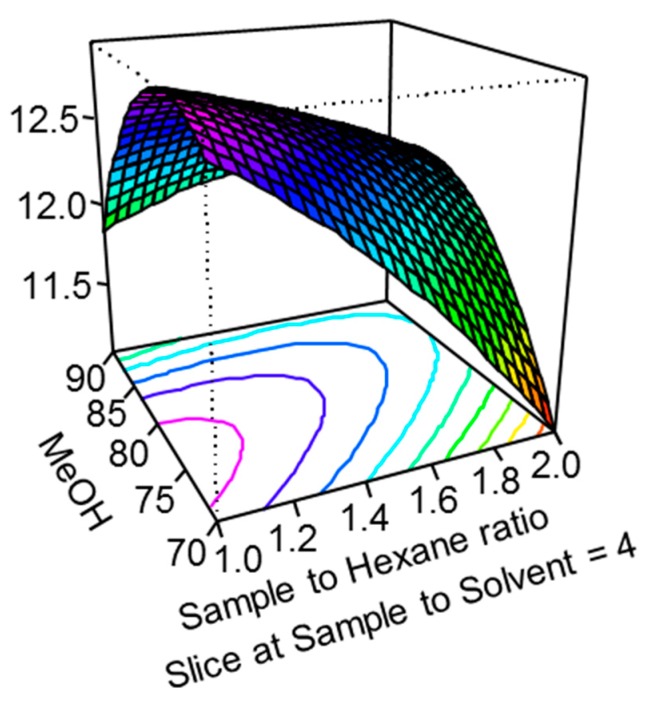
Response surface of TEN: slice at sample to extraction solvent ratio of 4.0.

Contrary to the vegetable-, fruit- or cereal-based food samples investigated frequently for *Alternarias* earlier (Table S1), the sunflower oil is a very lipophilic matrix and needs a unique sample preparation approach. Therefore, an experimental design was carried out to obtain the appropriate accuracy in different types of sunflower oil samples. A central composition design (CCD) has been planned with the statistical software R, version 3.0.2 for Windows. Two grams of the sample was used for sample extraction with methanol/water mixture; and *n*-hexane was applied for the elimination of lipophilic matrix constituents. This sample weight and these solvents have been found suitable for the *Alternaria* analysis (Table S1). The factors and levels were the following: (I) sample-to-hexane ratio: 1.0, 1.5, or 2.0; (II) methanol content in the extraction medium: 70%, 80%, or 90%; and (III) sample-to-extraction solvent ratio: 2.0, 4.0, or 6.0. A naturally contaminated sunflower oil was used for the experimental design that contained TEA (7.1 µg/kg) and TEN (12.8 µg/kg). An oil sample containing the toxins in much higher concentrations than LOQ would have been better for the CCD, but a sample with greater natural contamination could not be found. An optimal condition was achieved with the sample-to-hexane ratio of 1.0; 80% (*v*/*v*) methanol for extraction, and sample-to-extraction solvent ratio of 4.0 (Figure 3).

In this case, the sample dilution was 4-fold that increased the LOQ value, and AOH could not detected below 10 µg/kg with a signal-to-noise ratio (SNR) higher than 10. Therefore, an aliquot of methanolic extract (6 mL, equal to 1.5 g sample) was evaporated, and the final sample volume was adjusted to 0.5 mL with water that ended up with 3-fold sample pre-concentration (Section 4.2. and Section 4.3.). In this case, all compounds were detected with appropriate SNR at the desired levels. The other aim of sample evaporation was to lower the methanol content of the injection solution, and consequently, the deformation of TEA peak on the chromatogram could be avoided.

One aim of sample preparation is to reduce the ME of LC-MS/MS analysis [37]. ME is caused by the co-eluting matrix constituents and strongly influences the quantification [37]. The ME was studied with the optimal sample preparation conditions and was evaluated using the general approach [37]. Three matrix-matched calibrations were prepared from blank samples (i.e., three different sunflower oils). Blank extracts were spiked with standard mixture: the fortification levels were 10, 20, 30, 40, and 50 µg/kg for TEA, ALT, and TEN; and were 5, 10, 15, 20, and 25 µg/kg for AOH and AME. Low concentration levels were set due to the naturally low contamination of oils with these toxins reported in previous studies (see Table S1). AOH and AME are considered more toxic [4,6], therefore, twice lower levels were set for these two compounds. Calibrants in neat (matrix-free) solvent were also prepared and analyzed. The slopes of matrix-matched calibrations were compared to the slope of neat calibration in order to calculate the absolute ME. ME (%) = (slope in matrix-matched calibration/slope in neat calibration-1) × 100. ME < 0% means ion suppression, and ME > 0% indicates ion enhancement. The relative standard deviation (RSD%) of slopes obtained from the matrix-matched calibrations (*n* = 3) was calculated and evaluated as the relative ME [37]. Therefore, the relative ME means the precision of slopes in different matrix-matched calibrations.

The results obtained without ISTD correction indicated that the high matrix suppression influences the signal of AOH (20–48% ion suppression) and AME (75–88% ion suppression) (Table 2). For TEA, ALT, and TEN, a moderate ME could be seen. The relative ME was also considerable for AOH and AME (22–42%). The ME, however, could be compensated with isotope dilution (Table 2). The calibration evaluated by the ISTD method showed that the ME is greatly compensated, mainly for those two compounds (i.e., AOH and AME) that are considerably influenced by the co-eluting matrix constituents. The relative ME was also improved with ISTD correction. The high ion suppression for AOH and AME, however, indicated that considerable losses of the analytes occurred in the ion source. The reason for high ME is the remaining impurities (i.e., phospholipids) after extraction. Therefore, the SPE clean-up was tested for reducing the number and concentration of matrix constituents, which may lead to lower ME.

#### 2.2.2. Sample Preparation with Mixed-mode SPE Clean-Up

A sample clean-up utilizing mixed-mode SPE purification was tested. An aliquot (5 mL) of methanolic sample extracts (Section 4.2.) was diluted with 1% (*v*/*v*) acetic acid in water (35 mL) to lower the methanol content of the sample solvent. Diluted extracts were subjected to SPE clean-up (Section 4.4) using mixed-mode polymeric strong cation exchange cartridges (Strata-XL-C). Under acidic condition, this cartridge could selectively adsorb the toxins and basic matrix constituents on the reversed-phase and the cation exchange part of the cartridge, respectively [13]. Hence, the basic matrix solutes could be eliminated from the samples. The ME was studied after SPE clean-up. The SPE purification did not improve the absolute ME considerably. The signals of AOH and AME were still considerably suppressed (~ 50%) in the ion source, and only a slight improvement could be seen for AME (Table 2), however, the relative ME (14–17%) was significantly enhanced. This was an advantage of SPE clean-up, but the response correction with ISTD was also necessary after SPE clean-up. Again, the ISTD dilution could well compensate the ME (Table 2).

#### 2.2.3. Sample Preparation With Normal-Phase SPE Clean-Up

Oil samples can be easily dissolved in hexane that allow testing the normal-phase (NP) SPE clean-up (Section 4.5) with silica cartridges (Strata-Si-1). The washing solvent was a mixture of *n*-hexane and ethyl acetate, while the elution solvent was a mixture of methanol and acetonitrile. An optimization was based on an experimental design using CCD. The factors and levels were the following: (I) ethyl acetate content of the washing solvent: 10%, 20%, and 30% (*v*/*v*); (II) acetonitrile content of the elution solvent: 0%, 25%, and 50% (*v*/*v*); and (III) sample pre-concentration: 3-fold, 4-fold, and 6-fold. The same naturally contaminated sunflower oil was used for the experimental design mentioned above (12.8 µg/kg TEA and 7.1 µg/kg TEN). The results showed that there is no significant difference in concentrations obtained for TEN under different conditions. However, the NP SPE considerably lowered the accuracy of TEA. The recovery of TEA was around only 10% in all settings. The TEA could not be eluted from the silica cartridge with solvent containing only organic phase. Therefore, we added water into the elution solvent and tested the methanol/water mixture for sample elution with 10%, 20%, 30%, and 40% (*v*/*v*) water in methanol. Ten percent water in the elution solvent already resulted in ~ 70% recovery for TEA, which did not improve with a higher percentage of water. The drawback of having water in the elution solvent was that AME could not be eluted from the NP cartridge due to its lipophilic character. Hence, the NP SPE could not be used for all toxins involved.

### 2.3. Method Validation

The method was validated in accordance with the Commission Decision 2002/657/EC decision [38] and CEN/TR 16059:2016 guidelines [39]. The fortification levels were 10, 20, and 30 μg/kg for TEA, ALT, and TEN, respectively; and were 5, 10, and 15 for AOH and AME, respectively. These levels were set in line with the validation ranges used in the MVS [6]. Investigations at higher concentration levels were not needed because natural contamination of oils was reported at low µg/kg levels only. Six parallel samples were analyzed at each level that are in accordance with the EU guideline (Table S2). Measurements were carried out over 3 days, and all 54 samples were analyzed (3 levels × 6 samples × 3 days). The performance characteristics were as follows: selectivity, identification, linearity, recovery, precision, and limit of quantification (LOQ).

Blank samples were spiked and analyzed using the optimized method (Section 4.2 and Section 4.3). The chromatograms obtained from the blank samples were free of any interfering peak. For identification, the ion ratios (IAs) were calculated for all compounds in both neat standard solutions and samples. IAs were all within the tolerance ranges for all toxins (Table S2). The selectivity and identification met the criteria of EU guidelines [38]. Five-point calibration curves were performed to evaluate the linearity. Concentration levels, determination coefficients (R^2^), and equations are given in Table S2.

The requirement for recovery has been obtained between 70% and 120% at spiking levels used for validation [38,39]. The recovery varied from 73.6% to 95% at levels between 5 µg/kg and 15 µg/kg for AOH and AME. In the case of TEA, ALT, and TEN, the recovery was between 92.4% and 122% at the concentration range of 10–30 µg/kg. Only one value (122%) exceeded the acceptable ranges. Below the concentration of 100 µg/kg, the precision should be as low as possible [38], normally, RSD ≤ 30% [39]. The within-laboratory precision varied from 10.1% to 22.2% (Table S2). The LOQ was calculated from the SNR and evaluated as 10 times of SNR. The LOQ was checked by fortifying blank samples (*n* = 6) with standard solution to obtain the individual LOQ levels, and samples were analyzed. The SNR was above 10 in each sample and the IAs were in the acceptable ranges.

### 2.4. Analysis of Sunflower Oil Samples

Sixteen different brands and lots of sunflower oil samples were collected and analyzed for the five toxins mentioned above. Three samples were contaminated at low levels, in which only TEA and TEN were detected. One sample (cold pressed oil) contained both TEA (12.8 µg/kg) and TEN (7.1 µg/kg). The other two samples (refined oils) contained TEN at concentrations between 4.5 µg/kg and 5.0 µg/kg.

### 2.5. Analysis of Sunflower Seed QC Samples

In lack of sunflower oil QC samples, sunflower seed QC samples were measured. The method optimized for sunflower oil had to be modified to obtain the suitable method for sunflower seeds (Section 4.6). Both spiked (C08 SP and Q25 SP) and naturally contaminated (W52 NC) samples were tested. The samples were leftovers from MVS performed by JRC in 2018. The detected values and reference concentrations are given in Table S2. Even though the method presented herein was developed for sunflower oil samples, the concentrations detected in sunflower seed samples were not considerably different to the reference values. The method could not detect ALT at all, since the reference concentrations were all below the LOQ (10 µg/kg). Also, AOH and AME were not found in C08 SP due to the same reason. The accuracy of the method for sunflower seed samples was between 72% and 129% (Table S2).

## 3. Discussion

### 3.1. Method Development for LC-MS/MS Separation

The MS/MS detection of *Alternaria* toxins can be carried out in both positive and negative ionization modes (Table S1). The choice of polarization mode can be instrument dependent, but the negative mode usually results in a higher sensitivity for these toxins (Table S1) due to their weak acidic characteristics. We also observed considerable enhancement in sensitivity when negative ionization was applied. In addition to the ionization mode, the choice of ion source can also influence the sensitivity of MS/MS detection of *Alternarias*. Zwickel et al. [30] tested three ion sources (ESI, APCI, and atmospheric pressure photo ionization) for these toxins and found that the ESI was the most suitable one. In general, the ESI was employed (see Table S1), but Prelle et al. [16] reported three times higher responses for TEA when APCI was used, while the rest of the toxins had similar sensitivity in both ESI and APCI modes. Even though the ESI source of the applied LC-MS/MS instrument enabled appropriate sensitivity for the compounds other than *Alternarias*, the sensitivity for *Alternaria* toxins was quite a bit lower than those reported in earlier methods utilizing other types of instruments (Table S1). This led to the application of an APCI probe that significantly improved the instrumental LOQ for all compounds. One participant in the MVS 2018 used the same instrument as in our study and also applied the APCI source [7].

The mobile phase pH was set at 8.8 due to the negative ionization mode and the chromatographic separation of TEA. In the existing methods, the alkaline pH was used when the detection was carried out in negative ionization mode; and the acidic or neutral eluent pH was utilized if the positive ion mode or polarity switching was employed (Table S1). Even though the alkaline mobile phase pH is not usual in LC-MS/MS separation, it is feasible for *Alternaria* toxins due to the chromatographic problem with TEA at acidic pH condition. Moreover, the alkaline pH enhanced the sensitivity in the negative ion mode. The acidic pH condition did not result in the appropriate peak shape for TEA and also lowered the sensitivity of MS/MS detection in negative ionization mode. The chemical derivatization, suggested in some papers [13,19,20], was not tested. Although this approach enabled the simultaneous separation of *Alternaria* toxins [13], it could have further increased the LOQ and the preparation time and costs.

### 3.2. Method Development for Sample Preparation

In this study, we focused on sunflower oil samples only and developed a LC-ID-MS/MS method involving a unique sample preparation approach suitable for this kind of lipophilic matrix. The goal was to develop a dilute-and-shoot method that is frequently used in toxin analysis by LC-MS/MS method [40]. The non-polar matrix constituents of oil were eliminated with hexane that could easily dissolve the oil. The toxins have low solubility in hexane; hence, the target compounds could be extracted into a non-miscible solvent such as water, methanol or acetonitrile. Even though some studies have reported the use of the general acetonitrile-based mycotoxin extraction solvent mixture (acetonitrile/water/acetic or formic acid) [41] for *Alternarias* (Table S1), we did not prefer the acetonitrile as a solvent due the lower solubility of *Alternarias* in acetonitrile. Methanol is a more suitable solvent for these toxins, and therefore, methanol/water/acetic acid mixture has been utilized for extraction in the candidate method for standardization [6]. In the case of cereal samples, the extraction medium should contain water due to the starch content of samples; and the aqueous methanolic solvent in our case was needed to obtain better solvent separation between the hexane layer and the extraction medium. Also, water can enhance the extraction of TEA with polar characteristics. The experimental design showed that 80% (*v*/*v*) methanol in water gave the best extraction from the naturally contaminated oil. In other types of samples (e.g., tomato, cereals, and oilseeds), ~ 80% methanol also resulted in the optimal extraction solvent composition (Table S1). The HPLC separation was carried out at alkaline pH, so acid was not added into the extraction solvent to avoid the large pH difference between the injection solvent and the mobile phase, which could deform the chromatographic peak. The experimental design also indicated that the optimal sample-to-solvent ratio was 4.0, which is a general ratio in mycotoxin analysis based on the dilute-and-shoot approach [40,41].

High ME (mainly ion suppression) usually influences the mycotoxin analysis based on the LC-MS/MS method [40,41] that is also true for *Alternarias* [8,9,13,18,19,23,28,29,31,42]. The lower sensitivity of our instrument and the high ME for AOH and AME increased the LOQ. Hence, sample pre-concentration with evaporation and reconstitution was necessary to obtain appropriate LOQ (≤ 10 µg/kg) for all compounds. It should be pointed out that an instrument with higher sensitivity would allow further dilution of the extracts that could decrease the preparation time and the ME of analysis. The elimination of co-eluting matrix constituents was tested with SPE clean-up on mixed-mode cation exchange cartridges. Although the mixed-mode SPE and subsequent reversed-phase HPLC measurements enabled an orthogonal separation approach, considerable improvement in ME could not be seen (Table 2), and only the relative ME was enhanced. However, the sample preparation time and overall costs were also increased. In conclusion, the mixed-mode SPE clean-up did not improve the overall analytical process since it is time-consuming and more expensive compared to the dilute-and-shoot approach. The NP SPE clean-up was alternatively tested since this approach requires only sample dissolution in hexane and the dissolved samples can be directly subjected to NP SPE. The NP SPE clean-up would be a simpler clean-up approach, but using this process, we lost either the TEA or the AME, depending on the elution solvent composition. Overall, the dilute-and-shoot approach was the most suitable sample preparation method.

The need of isotope dilution for *Alternaria* toxin analysis by LC-MS/MS method has been strongly suggested by Asam and Rychlik [42] in 2015. Accordingly, isotopically labeled analogues were necessary for the analysis. In line with that, an important conclusion of the ME study was that all corresponding isotopically labeled analogues were necessary for the analysis. This study is the first in using all ISTDs for five *Alternarias* analyzed. While a moderate ion suppression influences the signal of TEA, ALT, and TEN, the ME for AOH and AME is much higher. This means that AOH-d3 and AME-d3 cannot compensate the ME of other analytes, and the ISTDs are not exchangeable. The differences in ME among the compounds analyzed can originate from the retention time differences between toxins and from the various structures of analytes. Even though ALT has similar structure to AOH, the 1.2 min of retention time difference (Figure 2) resulted in considerable difference in the ion suppression (Table 2). On the other hand, there was a significant difference in slopes of matrix-matched calibrations of AME obtained from three different oils. The relative ME was evaluated from the precision of slopes in the matrix-matched calibrations and showed that the matrix-matched calibration could strongly influence the quantification of AME. Hence, the isotope dilution method is needed for appropriate quantification. The relative ME values for AME were significantly improved with the ISTD evaluation (Table 2). In general, the relative ME values were improved for all compounds under ISTD evaluation. It means that the slopes of three different matrix-matched calibrations were close to each other, and they were nearly free of ME.

### 3.3. Real Sample Analysis

Chulze et al. [43] has already reported the high (30 µg/kg AME—15.796 µg/kg TEA) and frequent (85%) contamination of sunflower seeds with *Alternaria* toxins in 1995. Under processing sunflower oil from the oilseeds, the *Alternaria* toxins may appear in the oil product due to the contamination of sunflower seeds with these toxins. The high natural contamination of sunflower seeds with *Alternarias* reported recently worldwide [4,6,10,11,12] indicates that cross contamination with toxins can occur in the final sunflower oil products. Even though Chulze et al. [43] has described the decrease of *Alternaria* toxins during the processing of sunflower seeds to oil, the TEA and AME contamination of raw seeds were still detectable in lower concentrations in the oil after processing [43]. Due to the non-polar character of AME, the occurrence of this toxin in lipophilic oil matrix is more likely, as reported previously [43]. Accordingly, the polar characteristics of TEA inhibit its accumulation in oil that was also proven in another study [43], while the AOH contamination of oilseeds could not be detected in the oil product at all. It should be pointed out that the method used by Chulze et al. [43] had a LOQ of 50 µg/kg (AOH), but the recent methods have much lower analytical limits. To the best of our knowledge, no other newer studies have dealt with the decrease of *Alternaria* during the process of oil from sunflower seeds or other types of oilseeds. Since there is a great consumption of sunflower oil worldwide, the need for involving this sample in toxin analysis is to support the legislation. The analysis on sunflower oil was performed by Liu and Rychlik [8] in 2013. López et al. [11] has conducted studies involving other types of foods as well (Table S1). In 2016, López et al. [11] found relatively high (up to 1350 µg/kg) and frequent (80%) contamination of sunflower seeds with TEA, but sunflower oils contaminated with TEA above LOQ (5 µg/kg) were not found [11]. Only AME was detected at 17 µg/kg in one of 11 oil samples, and other toxins were all below the LOQ [11]. Liu and Rychlik [8] also investigated several types of refined and cold-pressed oil samples like pumpkin seed oil, rapeseed oil, sunflower oil, and thistle oil. They detected TEN in three refined (up to 3.95 µg/kg) and three cold-pressed (up to 6.73 µg/kg) sunflower oils, and also in a rapeseed cold pressed oil (up to 0.64 µg/kg). Furthermore, the dihydrotentoxin could be detected (up to 4.48 µg/kg) in three cold-pressed sunflower oil [8].

We have analyzed 16 sunflower oil samples: one sample was a cold pressed sample, and the others were refined ones. In agreement with Chulze et al. [43] and López et al. [11], AOH was not detected and a low concentration of TEA was found (12.8 µg/kg), but only in the cold pressed oil. In three samples, TEN was detected between 4.5 µg/kg and 7.1 µg/kg, similar to those reported by Liu and Rychlik [8]. We have also found that the cold-pressed oil is more likely to be contaminated than the refined samples. Comparatively, Chuzle et al. [43] did not investigate the TEN and López et al. [11] did not find TEN in sunflower oil. Both TEA and TEN have the least toxicity [4] and the detected concentrations are below the validation range suggested by CEN [6], therefore, these contaminations may not cause any risk to human health.

The focus of our study was on sunflower oil since ML would be set for sunflower in near future.

## 4. Materials and Methods

### 4.1. Standards, Reagents, Equipment, Samples

Tenuazonic acid (TEA), altenuene (ALT), alternariol (AOH), tentoxin (TEN), and alternariol monomethyl ether (AME) analytical standards were obtained from Romer Labs (Tulln, Austria) and individual 100 µg/mL stock solutions in methanol were prepared and then kept at −18 ^°^C for a year. The isotopically labeled analogues (ISTDs) were purchased from ASCA GmbH (Berlin, Germany). An ISTD mixture containing TEA-^13^C2 (2.5 µg/mL), ALT-d6 (1 µg/mL), AOH-d3 (0.5 µg/mL), TEN-d3 (0.5 µg/mL), and AME-d3 (0.5 µg/mL) in methanol was prepared and stored at −18 °C for a half year. Methanol, acetonitrile, *n*-hexane, ethyl acetate, ammonia (25%), acetic acid, and ammonium acetate were either of LC-MS or HPLC grade, purchased from the Merck-Sigma group (Schnelldorf, Germany). The PTFE syringe filters (13 mm, 0.45 µm), Strata-XL-C mixed-mode polymeric strong cation exchange SPE cartridges (3 mL, 200 mg), and Strata-Si-1 silica SPE cartridges (6 mL, 500 mg) were acquired from Gen-lab Ltd. (Budapest, Hungary). The LC-MS/MS analysis was carried out by an Agilent 1100 HPLC pump (Agilent; Waldbronn, Germany), which was coupled to an AB Sciex 3200 QTRAP triple quad MS detector, equipped with a Turbo Ion Spray APCI or ESI sources (Sciex; Warrington, Cheshire, UK). Data acquisition and evaluation were performed using the Analyst software version 1.5.2. (Sciex; Warrington, Cheshire, UK). Sample shaking and centrifugation were done using horizontal shaker SM 30 B (Edmund Bühler, Bodelshausen, Germany) and Jouan B4i centrifuge (Thermo Fisher Scientific, Budapest, Hungary), respectively. Sunflower oil samples of different brands and lots were purchased at local shops and originated from the EU. Three sunflower quality check (QC) samples (i.e., W54 NC, C08 SP, and Q25 SP) were leftovers from MVS organized by JRC in 2018. The stability of toxins in both food samples and sample extracts was studied by JRC during the MVS in 2016 and 2018 [6,7]. *Alternaria* toxins are stable at least up to 4 months in samples and they do not degrade in the autosampler in the aqueous injection solution during validation.

### 4.2. Sample Extraction

Sunflower oil samples (2.00 g) were weighed in polypropylene (PP) centrifuge tubes, followed by the addition of 2 mL *n*-hexane. The oils were completely dissolved in the tubes by vortex-mixing for 5 s. The sample-to-hexane ratio was 1.0. Then, 8 mL methanol–water (80/20, *v*/*v*) mixture was added to the samples and the tubes were capped, followed by vortex-mixing for 5 s. The sample-to-extraction solvent ratio was 4.0. Afterwards, the samples were shaken for 45 min at 180 rpm at ambient temperature. Then, the extracts were centrifuged at 4000× *g* for 2 min at ambient temperature and the hexane layer was discarded.

### 4.3. Sample Pre-Concentration

An aliquot (6.0 mL) of the extracts (equal to 1.5 g sample) was transferred into glass tubes and evaporated at 45 °C under a gentle stream of nitrogen to ~0.2 mL. Then, 50 µL ISTD mixture (Section 4.1) and water were added into the tubes to obtain 0.5 mL of volume, followed by sample reconstitution by vortex-mixing for 20 s. Finally, samples were filtered through the PTFE syringe filters into the HPLC vials and analyzed by the LC-ID-MS/MS method. In this case, the sample pre-concentration was 3-fold. The concentrations of ISTDs were: TEA-^13^C2 (83 µg/kg), ALT-d6 (33 µg/kg), AOH-d3 (17 µg/kg), TEN-d3 (17 µg/kg), and AME-d3 (17 µg/kg).

### 4.4. Sample Clean-Up on Mixed-Mode Cation Exchange Cartridges

An aliquot (5.0 mL) of the extracts was diluted with 35 mL 1% (*v*/*v*) acetic acid in water in new PP centrifuge tubes, which were capped. After homogenization by handshaking for 10 s, the diluted samples were subjected to SPE clean-up on Strata-XL-C cartridges (3 mL, 200 mg). Cartridges were conditioned with 3.0 mL methanol, followed by 3.0 mL water and 3.0 mL 1% (*v*/*v*) acetic acid in water. Diluted samples (40 mL) were passed the cartridges through the dropwise method. Then, cartridges were washed with 3.0 mL water, followed by 3.0 mL *n*-hexane. Afterwards, the cartridges were dried under vacuum for 1.0 min and the samples were eluted with 5.0 mL methanol into glass tubes. Samples were then evaporated and reconstituted as written in Section 4.3.

### 4.5. Sample Clean-Up on Silica Cartridges

Sunflower oil samples (3.00 g) were weighed into the PP centrifuge tubes, followed by the addition of 6.0 mL *n*-hexane. The oils were completely dissolved in the tubes by vortex-mixing for 5 s. Samples were subjected to SPE clean-up on Strata-Si-1 cartridges (6 mL, 500 mg). Cartridges were conditioned with 6.0 mL methanol, followed by 6.0 mL *n*-hexane. The samples dissolved in *n*-hexane were passed the cartridges through dropwise method. Then, cartridges were washed with 6.0 mL *n*-hexane. Afterwards, the cartridges were dried under vacuum for 1 min and samples were eluted with 6.0 mL methanol into glass tubes. Samples were then evaporated and reconstituted as written above.

### 4.6. Sample Preparation for Sunflower Seed Samples

Sunflower seed QC samples (2.00 g) were extracted with 8 mL methanol/water (80/20, *v*/*v*) mixture, then the extracts were de-fattened with 2.0 mL *n*-hexane (Section 4.2). The hexane layer was discarded, and 0.5 mL extract was diluted with ISTD mixture (50.0 µL). Afterwards, extracts were filtered through the PTFE syringe filters into HPLC vials and analyzed by LC-ID-MS/MS.

### 4.7. LC-ID-MS/MS Separation

Toxins were separated on a Zorbax Extend C-18 (150 mm × 3 mm, 5 µm) HPLC column (Agilent; Waldbronn, Germany) using a binary gradient elution. Solvent A contained 5.0 mM ammonium acetate in water (pH adjusted to 8.8 with ammonium hydroxide) and solvent B was methanol. The mobile phase consisted of 10% B at 0 min; 10% B at 1 min; 100% B at 10 min; 100% B at 14.0 min; 10% B at 14.1 min. Stop time was 21 min. The flow rate was 0.5 mL/min. The column thermostat was maintained at 30 °C. The injection volume was 10.0 µL. Compounds were detected in APCI negative ionization mode and using multiple reaction monitoring (MRM) scan type in the triple quadrupole MS/MS instrument. The ion transitions are given in Table 1. The LC-ID-MS/MS analysis was carried out using all corresponding isotopically labeled ISTDs. The ISTDs were employed to compensate the signal suppression/enhancement in the ion source (matrix effect) caused by the co-eluting matrix constituents.

During the method development, the positive ionization mode (Table 1) with both APCI and ESI sources and the ESI negative ionization mode were also tested. When the positive ionization was employed in the ion source, the separation was performed with 0.3% (*v*/*v*) acetic acid in water (mobile phase A) and 0.3% (*v*/*v*) acetic acid in methanol (mobile phase B) eluent composition using the same gradient elution as written above.

The ion source settings were as follows: nebulizer current (only with APCI ion source): −4 (negative ion mode) or 4 (positive ion mode); drying gas temperature: 600 °C; nebuliser pressure: 30 unit; drying gas flow: 30 unit; curtain gas: 20 unit; capillary voltage: −4200 V (negative ion mode) or + 5000 V (positive ion mode); collision gas (N_2_): medium unit; interface heater: on.

The optimal ion transitions are given in Table 1, and the optimal ionization mode was APCI negative. These detection parameters were used during validation and real sample analysis.

## Figures and Tables

**Table 1:** MS/MS detection parameters for *Alternaria* toxins detected in APCI and ESI ionization modes employing negative or positive ion mode. The quantifier ion transition is highlighted with bold.

Compounds	Ionization Mode	Precursor Ion (*m*/*z*)	Product Ion (*m*/*z*)	Dwell Time (ms)	Declustering Potential (V)	Entrance Potential (V)	Cell Exit Potential (V)	Collision Energy (V)	Collision Cell Exit Potential (V)
TEA	negative	196	83	50	−70	−9	−12	−32	0
			**139**	50				−26	0
TEA-^13^C2		198	**141**	50	−70	−9	−12	−26	0
ALT		291	161	50	−80	−10	−22	−52	0
			**203**	50				−40	0
ALT-d6		296	**203**	50	−80	−10	−22	−40	0
AOH		257	**147**	50	−65	−6	−22	−46	0
			213	50				−30	−5
AOH-d3		260	**218**	50	−65	−6	−22	−30	−5
TEN		413	141	50	−80	−5	−14	−28	0
			**271**	50				−20	−2
TEN-d3		416	**274**	50	−80	−5	−14	−20	−2
AME		271	228	50	−60	−2	−16	−36	−2
			**256**	50				−30	−2
AME-d3		274	**259**	50	−60	−2	−16	−30	−2
TEA	positive	198	139	50	66	10	12	19	4
			**153**	50				17	4
TEA-^13^C2		200	**155**	50	66	10	12	17	4
ALT		293	139	50	61	12	16	79	4
			**257**	50				19	4
ALT-d6		299	**262**	50	61	12	16	19	4
AOH		259	**128**	50	116	9	14	57	4
			185	50				14	4
AOH-d3		262	**131**	50	116	9	14	57	4
TEN		415	119	50	91	8	20	23	4
			**132**	50				53	4
TEN-d3		418	**135**	50	91	8	20	53	4
AME		273	115	50	126	9	16	69	4
			**128**	50				71	4
AME-d3		276	**131**	50	126	9	16	71	4

**Table 2:** The matrix effect (ME%) and relative matrix effect (RSD% of slopes) evaluated under different sample preparation and evaluation conditions. ME% < 0 means ion suppression, and ME% > 0 means ion enhancement.

	TEA	ALT	AOH	TEN	AME
	Preparation without SPE clean-up and evaluation without ISTD correction				
ME% (sample 1)	8	−7	−20	4	−75
ME% (sample 2)	−3	−3	−40	−13	−86
ME% (sample 3)	−5	−18	−48	−14	−88
Relative ME%	7	9	22	11	42
	Preparation without SPE clean-up and evaluation with ISTD correction				
ME% (sample 1)	11	−4	11	10	−6
ME% (sample 2)	4	−6	10	0	−11
ME% (sample 3)	5	−19	31	2	5
Relative ME%	4	9	10	5	5
	Preparation with mixed-mode SPE clean-up and evaluation without ISTD correction				
ME% (sample 1)	2	10	−45	10	−57
ME% (sample 2)	−10	−8	−57	−15	−50
ME% (sample 3)	−13	3	−46	−18	−62
Relative ME%	9	11	14	17	14
	Preparation with mixed-mode SPE clean-up and evaluation with ISTD correction				
ME% (sample 1)	8	1	2	9	6
ME% (sample 2)	−6	−7	4	−12	−3
ME% (sample 3)	−6	3	−18	−4	−8
Relative ME%	8	5	13	11	7

## Supplementary Materials

The following are available online. Table S1: Existing LC-MS/MS methods for *Alternaria* toxins; Table S2: The validation results for Alternaria toxins in sunflower oil samples and the results of analyzing sunflower seed QC samples by the modified method.
