# Supplementary material for: Determination of Alternaria Toxins in Sunflower Oil by Liquid Chromatography Isotope Dilution Tandem Mass Spectrometry
Source: Molecules. 2020 Apr 7;25(7):1685. doi: 10.3390/molecules25071685 (PMC7180471; doi:10.3390/molecules25071685)
Supplement: Supplementary file 1 [file molecules-25-01685-s001.pdf]

Supplementary Materials

Article

Determination of *Alternaria* Toxins in Sunflower Oil by Liquid Chromatography Isotope Dilution Tandem Mass Spectrometry

Ádám Tölgyesi [1,*], Luca Kozma [1] and Virender K. Sharma [2,*]

[1] KERMI department, ÉMI-TÜV SÜD Ltd., Dugonics utca 11, 1043 Budapest, Hungary; kozma.luca@emi-tuv.hu

[2] Program for the Environment and Sustainability, Department of Environmental and Occupational Health, School of Public Health, Texas A&M University, 212 Adriance Lab Rd., 1266 TAMU, College Station, TX 77843, USA

* Correspondence:tolgyesi.adam@emi-tuv.hu (Á.T.); vsharma@tamu.edu (V.K.S.)

Academic Editor: Ping-Chung Kuo

Received: 27 February 2020; Accepted: 3 April 2020; Published: date

Table S1. Existing LC-MS/MS methods for *Alternaria* toxins.

**Compounds**	**Matrix**	**Method**	**Isotope dilution**	**Extraction solvent**	**Clean-up**	**Analytical limit**	Reference
**TEA, ALT, AOH, TEN, AME**	**Tomato**	**LC-ESI-MS/MS, negative ionization, chemical derivatization, C-18 column, pH 3**	**No**	**MeOH**	**SPE**	**LOQ: 2-20 µg/kg**	[11]
ALT, AOH, AME	Garlic, dietary supplements	LC-ESI-MS/MS, positive ionization, C-18 column, pH 3	No	Ethyl acetate/acetic acid (95/5, v/v)	SPE	LOQ: 6-100 µg/kg	[12]
AOH, AME	Wine, beverages	LC-ESI-MS/MS, negative ionization, C-18 column, pH 7	No	Not performed	SPE	LOD: 0.01 µg/kg	[13]
TEA, ALT, AOH, TEN, AME	Beverages, tomato, basil, olive	LC-ESI-MS/MS, polarity switching, C-18 column, pH 7	No	MeOH	SPE	LOQ: 0.7 - 63 µg/kg	[14]
AOH, AME	Tangerine	LC-ESI-MS/MS, negative ionization, C-18 column, pH 7	No	AcN	SPE	LOQ: 0.5 µg/kg	[15]

AOH, AME	Wine, beverages	LC-ESI-MS/MS, negative ionization, C-18 column, pH 7	AOH-d4, AME-d4	Not performed	SPE	LOQ: 0.03-0.09 µg/kg	[16]
TEA	Tomato based foodstuff	LC-ESI-MS/MS, negative ionization, chemical derivatization, C-18 column, pH 7	TEA-^{13}C6-^{15}N	Ethyl acetate	SPE	LOQ: 0.3 µg/kg	[17]
TEA	Cereals	LC-ESI-MS/MS, positive ionization, chemical derivatization, C-18 column, pH 3	No	Ethyl acetate	Not performed	LOQ: 50 µg/kg	[18]
TEA, ALT, AOH, TEN, AME	Cereals method1	LC-ESI-MS/MS polarity switching, HSS-T3 column, pH 3	No	AcN + 1% (v/v) acetic acid	Not performed	LOQ: 2 µg/kg	[8]
TEA, ALT, AOH, TEN, AME	Wine, beverages, cereals method2	LC-ESI-MS/MS polarity switching, HSS-T3 column, pH 3	No	AcN/water/formic acid, 84/16/1 (v/v/v)	Not performed	LOQ: 1.0-5.0 µg/kg	[8]
TEA, ALT, AOH, TEN, AME	Sunflower oil, sunflower seeds, tomato based foodstuff	LC-ESI-MS/MS polarity switching,	No	AcN/water/formic acid, 84/16/1 (v/v/v)	Not performed	LOQ: 1.5-5.0 µg/kg	[9]

		HSS-T3 column, pH 3					
TEA, ALT, AOH, TEN, AME	Fig, sunflower seeds	LC-ESI-MS/MS polarity switching, HSS-T3 column, pH 3	No	AcN + 1% (v/v) acetic acid	Not performed	LOQ: 1.5-5.0 µg/kg	[9]
TEA, ALT, AOH, TEN, AME	Tomato, cereals	LC-ESI-MS/MS, negative ionization, C-18 column, pH 8.7	No	MeOH/water/acetic acid, 79/20/1 (v/v/v) hexane	Not performed	LOQ: 0.06 - 3.6 µg/kg	[19]
TEA, ALT, AOH, AME	Spices, herbs	LC-ESI-MS/MS, negative ionization, C-18 column, pH 8	No	AcN/water/acetic acid, 49/50/1 (v/v/v)	Not performed	LOQ: 4.2 - 20.4 µg/kg	[20]
TEA, AOH, TEN, AME	Cereals, tomato and fruit based samples	LC-ESI-MS/MS, negative ionization, C-18 column, pH 7	TEA-^{13}C6-^{15}N, AOH-d4, AME-d4	ACN/water, 84/16 (v/v/v) + 1,3% formic acid	SPE	LOQ: 0.16 - 5.56 µg/kg	[21]
TEA, AOH, TEN, AME	tomato and fruit based samples	LC-ESI-MS/MS positive ionization, phenyl-hexyl column	No	AcN + 1% (v/v) formic acid	QuEChERS	LOQ: 4.0 - 160 µg/kg	[22]
TEA, AOH, TEN, AME	Vegetables, fruits	LC-ESI-MS/MS polarity switching, C-18 column, pH 7	No	AcN + 1% (v/v) formic acid	QuEChERS	LOQ: 0.2 – 10 µg/kg	[23]

TEA, AOH, TEN, AME	Korean barley grain samples, rice culture medium extract	LC-ESI-MS/MS, positive ionization, C-8 column, pH 3	No	MeOH	Not performed	LOQ: 0.25 - 8.0 µg/kg	[24]
ALT, AOH, AME,	Sorghum	LC-ESI-MS/MS, positive ionization, C-18 column, pH 3	No	MeOH/ethyl acetate/water, 70/20/10, (v/v/v)	SPE	LOQ: 6.0 – 25 µg/kg	[25]
TEA, ALT, AOH, TEN, AME, conjugates	Tomato, fruit, vegetables	LC-ESI-MS/MS, polarity switching, HSS T3 column, pH 3	TEA-^{13}C6-^{15}N, AME-d4	AcN	QuEChERS	LOQ: 1.1 – 61.5 µg/kg	[26]
TEA, ALT, AOH, TEN, AME, conjugates	cereal-based foodstuffs	LC-ESI-MS/MS, polarity switching, HSS T3 column, pH 3	TEA-^{13}C6-^{15}N, AME-d4	AcN/water/acetic acid, 79/19.5/1.5, (v/v/v)	Not performed	LOQ: 0.9 – 8.3 µg/kg	[27]
TEA, ALT, AOH, TEN, AME and other toxins	wine, vegetable juices and fruit juices	LC-ESI-MS/MS, negative ionization, C-18 column, pH 8.8	No	Dilution with sodium hydrogen carbonate buffer, pH 8.4	SPE	LOQ: 0.4 – 3.1 µg/kg	[28]
AOH, AME	Foodstuffs	LC-ESI-MS/MS, negative ionization, C-18 column, pH 8.8	AOH-d4, AME-d4	AcN	MIP	LOQ: 0.6 - 1.8 µg/kg	[29]
TEA, ALT, AOH, TEN, AME and other toxins	Tomato products, bakery products, sunflower seeds,	LC-ESI-MS/MS, polarity switching,	No	AcN/water/formic acid, ratio depends on the matrix of interest	Not performed	LOQ: 0.1 - 110 µg/kg	[10]

	fruit juices, and vegetable oils	modified C-18 column, pH 3					
AOH, TEN, AME	Tomato, tomato-based products	LC-ESI-MS/MS, positive ionization, C-18 column, pH 3	No	AcN	Dispersive liquid-liquid microextraction	LOQ: 1.8 - 3.5 μg/kg	[30]
TEA, AOH, TEN, AME	Tomato, ketchup, fruit juices	LC-ESI-MS/MS, negative ionization, C-18 column, pH 8.8	No	AcN/water/MeOH, 45/45/10, (v/v/v)	SPE	LOQ: 0.3 - 20 μg/kg	[31]
TEA, ALT, AOH, TEN, AME and other toxins	Dried fruits	LC-ESI-MS/MS, polarity switching, modified C-18 column, pH 7	No	AcN	SPE	LOQ: 0.1 - 5 μg/kg	[32]
TEA, AOH, TEN, AME	Jujube	LC-ESI-MS/MS, positive ionization, C-18 column, pH 3	No	AcN + 1% (v/v) acetic acid	QuEChERS	LOQ: 0.5 - 0.9 μg/kg	[33]
TEA, AOH, TEN, AME	*Pennisetum glaucum*	LC-ESI-MS/MS, polarity switching, C-18 column, pH 3	No	AcN/water/acetic acid, 79/20/1, (v/v/v)	Not performed	No data	[34]
TEA, ALT, AOH, TEN, AME	Sunflower oil	LC-APCI-MS/MS, negative ionization, C-18 column, pH 8.8	TEA-^{13}C2, ALT-d6, AOH-d3, TEN-d3, AME-d3	MeOH/water, 80/20 (v/v), hexane	Not performed	LOQ: 2.5 – 10 μg/kg	Present study

TEA: tenuazonic acid; ALT: altenuene; AOH: alternariol; TEN: tentoxin; AME: alternariol monomethyl ether; AcN: acetonitrile; MeOH: methanol; MIP: molecularly imprinted polymer; LOQ: limit of quantification; LOD: limit of detection; SPE: solid phase extraction; QuEChERS: quick, easy, cheap, effective, rugged, and safe.
